# Developmental expression of “germline”- and “sex determination”-related genes in the ctenophore *Mnemiopsisleidyi*

**DOI:** 10.1186/s13227-016-0051-9

**Published:** 2016-08-02

**Authors:** Adam M. Reitzel, Kevin Pang, Mark Q. Martindale

**Affiliations:** 1 Department of Biological Sciences, University of North Carolina at Charlotte, 9201 University City Blvd., Charlotte, NC USA; 2 Sars International Centre for Marine Molecular Biology, Thormøhlensgt. 55, 5008 Bergen, Norway; 3 Whitney Laboratory for Marine Bioscience, University of Florida, St. Augustine, FL USA

**Keywords:** Cell proliferation, Dmrt, Germline, *Mnemiopsisleidyi*, Stem cell

## Abstract

**Background:**

An essential developmental pathway in sexually reproducing animals is the specification of germ cells and the differentiation of mature gametes, sperm and oocytes. The “germline” genes vasa, nanos and piwi are commonly identified in primordial germ cells, suggesting a molecular signature for the germline throughout animals. However, these genes are also expressed in a diverse set of somatic stem cells throughout the animal kingdom leaving open significant questions for whether they are required for germline specification. Similarly, members of the Dmrt gene family are essential components regulating sex determination and differentiation in bilaterian animals, but the functions of these transcription factors, including potential roles in sex determination, in early diverging animals remain unknown. The phylogenetic position of ctenophores and the genome sequence of the lobate *Mnemiopsisleidyi* motivated us to determine the compliment of these gene families in this species and determine expression patterns during development.

**Results:**

Our phylogenetic analyses of the vasa, piwi and nanos gene families show that *Mnemiopsis* has multiple genes in each family with multiple lineage-specific paralogs. Expression domains of *Mnemiopsis* nanos, vasa and piwi, during embryogenesis from fertilization to the cydippid stage, were diverse, with little overlapping expression and no or little expression in what we think are the germ cells or gametogenic regions. piwi paralogs in *Mnemiopsis* had distinct expression domains in the ectoderm during development. We observed overlapping expression domains in the apical organ and tentacle apparatus of the cydippid for a subset of “germline genes,” which are areas of high cell proliferation, suggesting that these genes are involved with “stem cell” specification and maintenance. Similarly, the five Dmrt genes show diverse non-overlapping expression domains, with no clear evidence for expression in future gametogenic regions of the adult. We also report on splice variants for two *Mnemiopsis* Dmrt genes that impact the presence and composition of the DM DNA binding domain for these transcription factors.

**Conclusion:**

Our results are consistent with a potential role for vasa, piwi and nanos genes in the specification or maintenance of somatic stem cell populations during development in *Mnemiopsis*. These results are similar to previous results in the tentaculate ctenophore *Pleurobrachia*, with the exception that these genes were also expressed in gonads and developing gametes of adult *Pleurobrachia*. These differences suggest that the *Mnemiopsis* germline is either specified later in development than hypothesized, the germline undergoes extensive migration, or the germline does not express these classic molecular markers. Our results highlight the utility of comparing expression of orthologous genes across multiple species. We provide the first description of Dmrt expression in a ctenophore, which indicates that Dmrt genes are expressed in distinct structures and regions during development but not in future gametogenic regions, the only sex-specific structure for this hermaphroditic species.

**Electronic supplementary material:**

The online version of this article (doi:10.1186/s13227-016-0051-9) contains supplementary material, which is available to authorized users.

## Background

Sexual reproduction is critical for nearly all animals as it is the means by which individuals pass on their genetic material to future generations. Thus, the specification and development of gametes in males and females is a central process in animal life cycles. Due to the commonality of sexual reproduction, we may expect that deeply conserved molecular mechanisms may be responsible for these developmental events in all animals. However, despite the presence of sperm and oocytes in all sexually reproducing animals, the relative conservation of the mechanisms responsible for their specification and differentiation remains debated. The uncertainty about the extent of homologous mechanisms for developmental processes related to sexual reproduction results, in part, from the diversity of described processes in various lineages but also from the relative paucity of species that have been studied. Research with species from early diverging animal phyla, including Porifera, Cnidaria and Ctenophora, has proven insightful for comparative research describing the origin of gene families central to particular developmental processes in bilaterians as well as the conservation of function in deep animal evolution. Two general developmental processes, for which studies in these lineages could be insightful, are related to sexual reproduction in animals: (1) specification of a set of cells for the germline and (2) differentiation of the germline into the sex-specific gametes, sperm and oocytes, as part of the sex determination in the reproductive adult. Studying these two related questions in early diverging phyla is important to inform two general research areas in comparative evolutionary developmental biology: What is the potential conservation compared with bilaterian species, and what functions do molecules commonly used in germline specification and gamete differentiation have in other developmental processes.

### Germline specification

The precursors to the gametes, referred to as the germ cells and part of the germline, become distinct from somatic cell lineages during development, but the timing of this transition from somatic to germline appears labile over animal evolution [1–3]. The germline in animals is specified via two general processes: preformation and epigenesis [reviewed in 3]. Preformation occurs when maternally synthesized cytoplasmic proteins and mRNAs (i.e., germ plasm) are deposited and segregated in the oocyte such that the blastomeres that inherit them become primordial germ cells (PGCs), which later differentiate into germ cells [4]. Ectopic expression of these determinants will cause other somatic cells to become germ cells, and removal or inhibition results in loss of the germline. Preformation is the mechanism of germline determination in traditional invertebrate models such as *Drosophilamelanogaster* and *C. elegans* as well as many vertebrates. Epigenesis is the alternate mode of germline specification where there is no maternally deposited germ plasm and germ cells are determined later in development via inductive interactions between cells. Here, a population of cells with the potential to become germ cells are induced by a signal to differentiate into PGCs. Epigenesis is particularly common and well studied in mammals [5]. Across the metazoa, preformation is more widely understood due to its commonality in traditional model organisms; however, epigenesis is likely more common and widespread across the animal kingdom and likely the ancestral condition of the stem animal [3]. Species in early diverging phyla in most all cases have germ cell development consistent with epigenetic mechanisms [but see 6 that suggest preformation may date to cnidarian–bilaterian ancestor], but the mechanisms remain largely unstudied.

While germ cells were originally characterized morphologically by a large round nucleus, large nucleolus, relatively clear cytoplasm and granular cytoplasmic material, often referred to as “nuage” [reviewed in 3], in recent years, molecular markers for protein and RNA expression have provided a useful way of identifying germ cells [7–9]. Three prominent genes have been characterized showing a conserved expression pattern in germ cells: nanos, vasa and piwi [10]. nanos is a CCHC zinc finger protein that likely functions as a transcriptional and translational repressor. Originally isolated from *Drosophila*, nanos is an important regulator involved with both anterior–posterior patterning as well as germ cell specification [11, 12]. Similarly in zebrafish, uniform maternal *nanos* transcripts later become concentrated in the PGCs [13]. In the sea urchin, *Strongylocentrotus purpuratus*, nanos is involved in oogenesis and then later localized only to the small micromeres [14], which give rise to germ cells [15, 16]. In the annelid *Capitella teleta*, it is expressed maternally and through cleavage in a uniform pattern, later becoming segregated to both putative PGCs and a subset of somatic cells [17]. Similar maternal and then segregated expression of *nanos* in subsets of blastomeres has been reported in molluscs [18]. Work in three cnidarians, *Nematostella*, *Clytia* and *Hydra*, has shown expression of nanos paralogs in putative germ cells, stem cells, as well as somatic cells (e.g., base of tentacle bulbs in *Clytia*) [6, 19, 20]. nanos is expressed in the oocytes and during development of the sponge *Sycon ciliatum* [21].

Another widely studied germ cell marker is the DEAD box RNA helicase vasa [22, 23]. In *Drosophila*, this gene, involved in germ plasm assembly and translational regulation, is expressed maternally early, later in the germ cells and finally in both the ovary and testes [reviewed in 4]. It is expressed in the PGCs or putative PGCs in zebrafish [9], *Xenopus* [24, 25], *Capitella* [17], *Strongylocentrotus* [14], as well as other bilaterians. In cnidarians, vasa is expressed in a variety of tissues. In *Hydra*, it is expressed in the germline, interstitial stem cells and the ectoderm [20]. Similarly, in the hydrozoan *Clytiavasa* is expressed in the gonad of males and females, stem cells of the medusa, maternally in developing oocytes and zygotically during embryogenesis, potentially for specification of interstitial stem cells [6]. However, in another hydrozoan *Hydractinia*, it is expressed in interstitial stem cells in the endoderm during gastrulation, in the ectoderm at metamorphosis and interstitial stem cells in polyp stages but not in the gonads [26]. However, studies examining the protein distribution show localization to possible germ cells and stem cells during polyp stages. In the anthozoan *Nematostella*, vasa paralogs are expressed endodermally and then in putative germ cells [19]. vasa has also been shown to be expressed in oocytes and developing larvae for the sponge *Sycon* [21].

piwi is another gene commonly used as a germ cell, as well as stem cell marker. Originally named in *Drosophila* for P-element-induced wimpy testis, this gene is involved in maintenance of stem cells, as well as controlling cell division rates [27]. piwi is also involved in the repression of retrotransposons and RNA interference pathway. These argonaute-like proteins form a complex with a specialized class of miRNAs, termed piRNAs for piwi-interacting RNAs, to degrade specifically targeted mRNAs, thereby preventing their translation [28–30]. piwi is expressed in germ cells, stem cells, as well as somatic cells in many animals [31–37], including cnidarians [6, 38] and the sponge *Ephydatiafluviatilis* [39].

### Sex differentiation

The specification of a subset of cells to the germline places these cells on a separate developmental trajectory as relatively undifferentiated stem cells that will eventually undergo meiosis and give rise only to the gametes. Differentiation of sperm or oocytes in males and females, respectively, routinely relies on cell–cell communication between the germ cell and surrounding somatic cells. The Dmrt (doublesex/mab-3-related transcription factor) gene family is the only group of genes identified to have a conserved role in somatic reproductive system, especially sex differentiation of the gonadal tissue development, in lineages across the bilaterians [40, 41]. In many metazoan phyla including arthropods [42–44], molluscs [45] and vertebrates [46], a subset of DMRT proteins in these species are involved in regulating sexual determination and/or differentiation, most of which promote male phenotypes. These data suggest that members of the Dmrt family may have been involved in differentiation of male-specific gametogenic tissues in the ancestor to bilaterians. Whether DMRT proteins played a role in development of gametogenic regions earlier in animal evolution is unclear. The Dmrt family is restricted to animals, and genes have been reported in all phyla, with the potential exception of Porifera [47]. Diversity and expression of Dmrt genes have been reported in two cnidarians, *Acroporamillepora* [48] and *Nematostellavectensis* [49], where expression was associated with times of reproduction in the coral and expression varied with development stage, tissue and sex in the sea anemone. Phylogenetic analyses of cnidarian Dmrt genes show little to no orthology with bilaterian subfamilies, obscuring a comparison of potential conserved functions for specific Dmrt genes at the cnidarian–bilaterian ancestor. While much of the focus on the Dmrt family has been related to sexual development and reproduction, research in various species has indicated that Dmrt genes have diverse roles in development and physiology [50–52], suggesting that functions other than sex differentiation may more likely be conserved in animals [53].

Ctenophores represent an informative lineage for examining the evolution of germ cells and molecular mechanisms of gamete differentiation. Besides their phylogenetic position as one of the earliest branching metazoans [54–57], they undergo unique reproductive and developmental strategies. All but one species (*Ocyropsis*) of ctenophores are self-fertile hermaphrodites, with separate male and female gonads located in the meridional canals beneath the comb rows [58]. Each comb row contains both ovaries and testes. The ovaries border the major planes, the tentacular and sagittal, while the testes are interradial and border the minor planes. Typically they can be identified both morphologically with the eye and histologically for identification of subcellular structures [58]. There are separate male and female gonoducts, although eggs are often fertilized as they are released. Lobate ctenophores, including *Mnemiopsis leidyi*, are unique in that they also undergo what has been called “dissogeny” or larval reproduction [58–60]. These species are capable of undergoing a reproductive period, while still a cydippid larva, however, only the subsagittal gonads become mature. While the function of dissogeny has been discussed, the exact cause is not known since young cydippids only periodically will release these gametes. After this early reproductive period, they later become reproductive again as juveniles and adults.

The origin of ctenophore germ cells is not clear. They are thought to originate from the endoderm, mainly because they are observed in association with the endoderm. Earlier speculations about an association with ectoderm were refuted [58]. Germ cells were identified morphologically as early as cydippid stages in three clusters, two male and one female, in the meridional canal primordia on each side of the cydippid. In later stages, the middle cluster, the female germ cells, splits and becomes divided by the meridional canal primordia. These germ clusters proliferate in the aboral direction, with the bands facing the tentacular plane forming testes and those facing the sagittal plane forming ovaries [61]. It is not known where these germ cells originate or exactly how the adult gonads form.

vasa and piwi expressions were previously studied in adults for another ctenophore species, the tentaculate *Pleurobrachiapileus* [62]. vasa and two piwi paralogs were expressed in the female and male germline, suggesting a potential role in either specification or maintenance of these gamete-producing cells. In addition, vasa and one piwi paralog (*PpiPiwi1*) as well as *Bruno*, and *PL10*, genes commonly associated with the germline in other animals, were expressed in presumptive stem cells associated with the exclusively somatic regions of the body, including the comb rows, tentacles and apical organ. Together, these results suggest that this set of “germline”-associated genes has a potentially conserved role in both germline specification/maintenance and stem cell maintenance dating to the ancestral animal [62, 63]. However, determination of the expression of these genes in development is also necessary to characterize when these genes are expressed and whether expression at particular early development stages can differentiate between the specification of the germline during embryogenesis or whether these genes are only expressed later in the adult. While multiple Dmrt genes have been reported in ctenophores [47], there are no reports of expression in any species of ctenophore.

We identified members of all the bilaterian “germline”- and “sex determination”-related gene families in the genome of *Mnemiopsisleidyi*, including nanos, vasa, piwi and Dmrt, as well as associated germline genes *Tudor*, *Bruno*, *Mago nashi* and *Pumilio*. In order to better understand the origin of the germ cells and specification of gamete-specific gametogenic regions, we examined the expression of the “germline” markers nanos, vasa, piwi and five members of the Dmrt gene family. We predicted that due to the early reproductive capabilities of *Mnemiopsis*, germ cells are specified relatively early in development and most likely originate from endodermal precursors. While the germ cells have been described to originate epigenetically, it remains possible that they could be preformed and migrate to their destination in the endoderm. Expression domains of nanos, vasa, piwi and Dmrts were diverse, with little overlapping expression during development, and no or little expression in what we think are the location for germ cells or gametogenic regions during these early developmental stages. Later in cydippids, we observed overlapping expression domains in the apical organ and tentacle apparatus for the “germline genes,” which are areas of high cell proliferation, suggesting that these genes may be more involved with “stem cell” specification and maintenance.

## Methods

### Gene identification and phylogenetic analyses

nanos, vasa (and PL10), piwi (and ago) and Dmrt genes (Additional file 1: Table S1) were identified via in silico searches of the *Mnemiopsis* sequence resources [Protein Models 2.2, 55]. A subset of nanos, vasa and Dmrt genes were additionally targeted for RACE PCR to determine full-length sequences and potential splice variants in a library produced from mRNA pooled from diverse developmental stages. RACE products (primers in Additional file 1: Table S1) were cloned into pGEMT (Promega) and sequenced. Overlapping fragments were assembled in silico to produce the complete transcript. To confirm the two gene products, we amplified and sequenced the entire open reading frame for each Dmrt gene. Each primer pair yielded a single product that matched the conceptually assembled fragment. Gene orthology was determined using MrBayes 3.2.5 [64, 65] using representative sequences for each gene family [19, 47, 62]. Full-length sequences for all taxa for each gene family were aligned with Muscle 3.6 [66] and edited manually in the case of clear errors. Four chains with 2 million generations were run using the LG substitution model, with trees sampled every 500 generations. The first 0.5 million generations were discarded as burn-in with a consensus generated using the remaining trees. Log-likelihood values were plotted and found to be asymptotic well before the burn-in fraction. Phylogenetic analyses describing the relationship of the 5 Dmrt genes from *Mnemiopsis* were recently published [47]. Our phylogenetic analyses resulted in similar ambiguous relationships for the ctenophore sequences relative to Dmrt genes from bilaterian species and cnidarians as reported by Wexler et al. [47]; thus, these phylogenies are not reported in this study. Trees were visualized and illustrated with FigTree v1.4 (http://tree.bio.ed.ac.uk/software/figtree/).

### Splice variants *MlDmrtA* and *B*

From our RACE PCR for the five *Mnemiopsis* Dmrt genes, we identified multiple bands in PCRs amplifying the 3′ end of transcript for two of them: *MlDmrtA* and *MlDmrtB*. Amplicons of different sizes using a “forward” primer located 68- and 91-bp downstream from the start site for *MlDmrtA* and *MlDmrtB*, respectively, were gel extracted, cloned and sequenced. To determine which exons composed each transcript, we characterized gene structure for each *Mnemiopsis* Dmrt splice variant by aligning the full-length transcripts to the assembled genome. In addition, we translated each splice variant, aligned to each other as well as the protein model from the *Mnemiopsis* genome database and compared presence/absence of the two conserved domains for Dmrt proteins: the DNA binding domain (DM domain) and the DMA domain. PCRs did not yield successful amplification of the 5′ end using the “reverse” primers for either *MlDmrtA* or *MlDmrtB*. Our PCRs also did not result in candidates for potential splice variants for the other Dmrt genes. However, we were not specifically exhaustively searching for splicing variants in this study and, thus, additional splice variants are possible.

### Developmental gene expression

*Mnemiopsis leidyi* adults were collected from Eel Pond or the NOAA Rock Jetty, Woods Hole, MA, USA, during the months of June and July and spawned as previously described [67]. For whole-mount in situ hybridization, embryos were fixed at various stages from fertilized oocytes (0 hpf) to cydippids (24–36 hpf). In situ hybridization was performed as described previously using an alkaline phosphatase-conjugated digoxigenin antibody (Roche Applied Science) and the substrates nitro-blue tetrazolium (NBT)/5-bromo-4-chloro-3-indolyl phosphate (BCIP) [68]. Cloned PCR products for riboprobe synthesis resulted from amplification of gene-specific primer combinations (primers in Additional file 1: Table S2). Gene expression was determined for oocytes and developmental stages from fertilization to cydippid stage, approximately 24 hours postfertilization (hpf). For fluorescent in situ hybridization, a similar protocol was used to generate digoxigenin and fluorescein-labeled RNA probes, with the following exceptions. Following hybridization and subsequent washes, endogenous peroxidase activity was quenched with a 30-min incubation in 1 % hydrogen peroxide. After blocking, embryos were incubated with peroxidase-conjugated anti-digoxigenin (diluted 1:500) or anti-fluorescein (diluted 1:200) antibodies overnight at 4 °C. Embryos were then washed with Tris-buffered saline plus Tween, and signals were detected with tyramide-conjugated Cy3 or FITC. Peroxidase activity was then quenched with a 2 % hydrogen peroxide incubation for 1 h, thereafter proceeding with the incubation of the second antibody.

### Cell proliferation and antibody staining

To measure cell proliferation, we used the Click-iT EdU labeling kit (Invitrogen, Molecular Probes). This kit incorporates EdU, a uridine analog, in cells that are undergoing S phase of mitosis. Embryos aged 12–24 h were incubated with the Edu labeling solution for 15–20 min and were subsequently fixed using 4 % paraformaldehyde with 0.02 % glutaraldehyde for 30 min. After three washes in PBS (phosphate-buffered saline), they were then stored in PBS at 4 °C until subsequent usage (less than 6 months). Prior to the Click-iT reaction, embryos were washed for 20 min in PBS plus 0.2 % Triton. The Click-iT reaction was performed according to the manufacturer’s instructions, utilizing the Alexa-488 reaction kit. To visualize nuclei, embryos were stained with Hoechst 33342 (Invitrogen, Molecular Probes). For counterstaining, some embryos were stained with anti-tyrosine tubulin (Sigma T9028) overnight at 4 °C. Antibody staining was performed following the Click-iT reaction. An Alexa-594-conjugated goat anti-mouse secondary antibody (Invitrogen, Molecular Probes) was used. Embryos were mounted in PBS and imaged under a Zeiss Axio Imager or LSM710 confocal microscope.

## Results

### Gene identification and phylogenetic analyses

BLAST similarity searches of the protein models and genome of *Mnemiopsis* resulted in matches for two nanos genes (*MlNanos1*, *MlNanos2*), six Vasa/PL-10 genes (*MlVasa1*–*5*, *MlPL10*) and five Piwi/Ago genes (*MlPiwi1*–*4*, *MlAgo*-*like*). We also identified other germ cell markers *Tudor*, *Pumilio* and *Bruno*. Consistent with Wexler et al. [47], we also recovered five *Dmrt* genes with unsupported relationships with other animal genes/subfamilies within the Dmrt family. Five of the identified vasa genes form a single clade with a previously identified vasa gene from *Pleurobrachia* (*PpiVasa*) within the vasa class of DEAD box RNA helicases (Additional file 2: Figure S1). The sixth vasa-like gene identified (Ml310319a) groups with *PleurobrachiaPL*-*10* in a well-supported group within the PL-10 class. We identified four piwi genes and a single *Argonaute*-like gene. Phylogenetic analyses (Additional file 2: Figure S2) show that there are two large classes of piwi genes, piwiA and piwiB [nomenclature after 69]. Most major animal lineages (cnidarians, sponges, deuterostomes, protostomes) possess genes belonging to both classes suggesting this family diverged early in metazoan evolution. One *Mnemiopsis* gene (*MlPiwi2*) groups with the piwiB class. The other three piwi genes form a separate clade with the two previously described piwi genes from *Pleurobrachia* (*PpiPiwi1* and *PpiPiwi2*). The ctenophore group of piwi genes forms a sister group to all other piwi genes from animals, including a clade of piwi genes restricted to arthropods. Clades of non-piwiA and piwiB genes have also been reported from planarians [69]. These ctenophore and protostome genes are potentially highly divergent, or else they may indicate further diversification of this gene family in each of these lineages. The two nanos genes from *Mnemiopsis* appear to be paralogs, although our phylogeny had overall very low resolution limiting an assessment of evolutionary relationships (Additional file 2: Figure S3).

### Splice variants for Dmrt genes

**Fig. 1:**
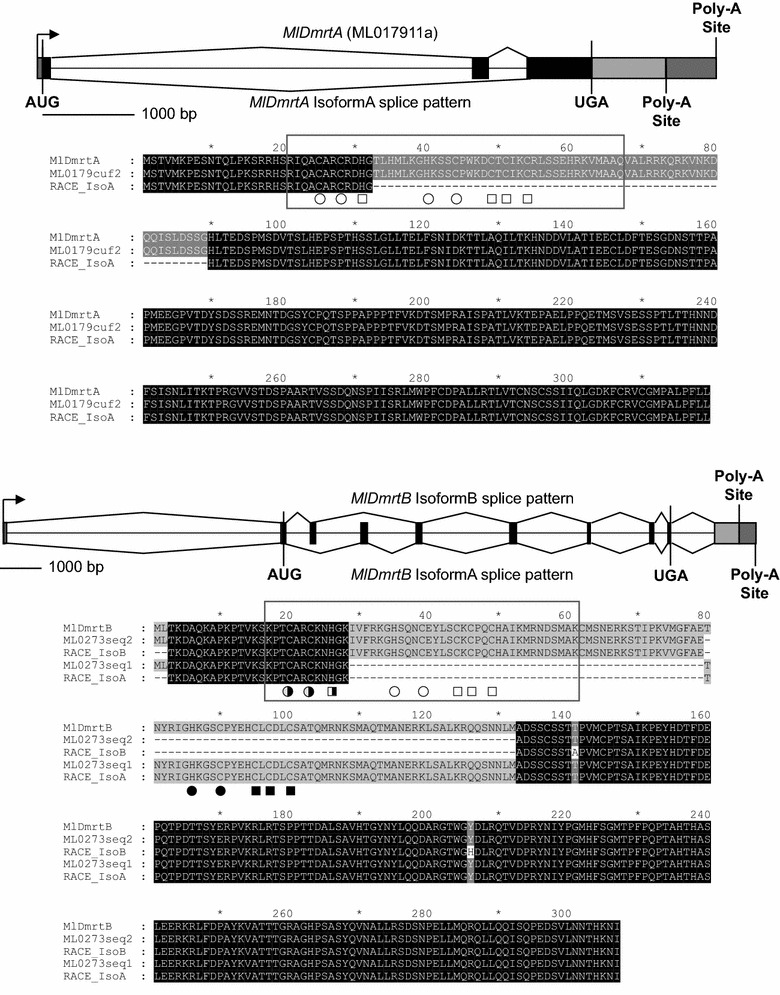
Splice variants for *MlDmrtA* and *MlDmrtB*. (*Top*) *MlDmrtA* is a three-exon gene with the start site located in the first exon, the DM DNA binding domain spanning exons 1 and 2 and the stop codon located in the third exon. The DM domain is indicated in the alignment with a *box* and the double zinc binding modules indicated by *circles* (Zn-binding module 1) and *squares* (Zn-binding module 2) below the alignment [regions defined as in 81]. We recovered a splice variant sharing the same start and stop positions but lacking exon 2 and thus the complete DM domain. The splice variant lacking exon 2 also had a shorter 3′ UTR indicated by a different polyadenylation site. (*Bottom*) *MlDmrtB* is a ten-exon gene locus. However, both our RACE sequencing and the assembled gene models (Trinity and Cufflinks) available at the *Mnemiopsis* genome database indicated two nine-exon genes, none with all ten exons. Both isoforms shared the same start and stop codons. IsoformA lacks exon 3, and IsoformB lacks exon 4. Contrary to the splice variant for *MlDmrtA*, both splice variants of *MlDmrtB* appear to have complete, but different, DM domains (indicated by *white* and *black symbols*), with a portion of each Zn-finger domain located in exon 2 and shared by both splice variants. The DM domain for IsoformB is indicated with a *box*. Like *MlDmrtA*, the two splice variants have different polyadenylation sites

RACE PCR with primers designed to amplify the 3′ end of each *Mnemiopsis* Dmrt gene resulted in single amplicons for *MlDmrtC*–*E* but two products for *MlDmrtA* and *MlDmrtB*. Alignment of these products to the *Mnemiopsis* genome revealed that these amplicons corresponded to splice variants for each gene. *MlDmrtA* is composed of three exons, and amino acids corresponding to the DM domain (DNA binding region) span the exon 1 and exon 2, with a portion of each of the two sets of zinc-chelating amino acids and DNA contact residues in each exon (Fig. 1). We searched the assembled transcripts available as part of the *Mnemiopsis* genome browser and identified a transcript in the Cufflinks assembly (ML0179_cuf_28) confirming all three exons and their sequence for the reference gene model (ML017911a). The splice variant (IsoformA) lacks exon 2 and thus has an incomplete DM domain lacking most of the zinc-chelating amino acids as well as amino acids likely to contact DNA. Our RACE products for each variant also show that each transcript has a different 3′ UTR, where the UTR for the larger transcript containing all exons is ~540 bp longer. The two amplicons from PCRs of *MlDmrtB* corresponded to splice variants, both of which lacked one of the 10 exons. IsoformA was a splice variant lacking exon 3, and IsoformB lacked exon 4. Like the splice variants for *MlDmrtA*, these two isoforms of *MlDmrtB* also had different 3′ UTRs, where the UTR for IsoformA was ~420 bp longer. Unlike *MlDmrtA*, the two splice variants for *MlDmrtB* appear to translate into proteins with complete, but different, DM domains. Like *MlDmrtA*, a portion of each zinc-chelating amino acid set is present in one exon (exon 2). For *MlDmrtB* IsoformA, the remaining residues are present and appropriately spaced in exon 4, while for IsoformB, the residues are in exon 3. We searched the assembled transcripts at the *Mnemiopsis* genome browser and identified matching sequences to our RACE products (IsoformA, ML0273|comp17369_c0_seq1; IsoformB, ML0273|comp17369_c0_seq2). A transcript containing all 10 exons was not identified in either our sequencing or in the transcripts assembled through the *Mnemiopsis* genome project.

### Developmental gene expression and regions of cell proliferation

**Fig. 2:**
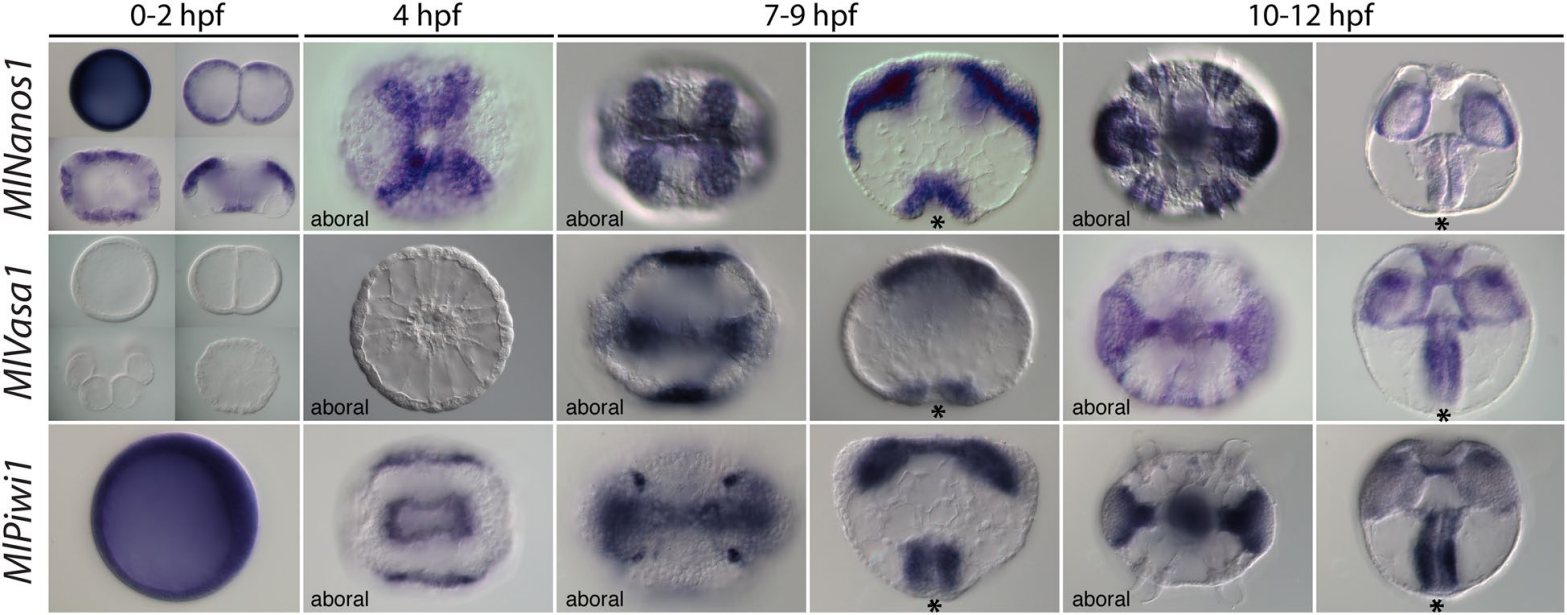
Expression of nanos, vasa and piwi homologs during *Mnemiopsis* development. All views are lateral, unless otherwise specified, with the asterisk marking the position of the blastopore or mouth. (*Top row*) *MlNanos1* was expressed uniformly in the egg and through cleavage stages and blastulae (0–2 hpf). From gastrulation (4 hpf) onward, expression was confined to the developing comb plates and pharynx. In later stages (10–12 hpf), there was also additional expression in the tentacle bulbs. (*Middle row*) *MlVasa1* was not detected until after gastrulation, at 7–9 hpf. It was expressed in the blastopore and eventually the pharynx. *MlVasa1* was also expressed in the ectoderm along the sagittal axis. Additionally, it was expressed in mesodermal derivatives. In later stages (10–12 hpf), expression was detected in the pharynx, tentacle bulbs and in the ectoderm between the comb rows. (*Bottom row*) *MlPiwi1* is expressed uniformly in the egg. At gastrulation, it was downregulated except for the invaginating pharynx, mesodermal derivatives and four other groups of cells. In later stages, it remained expressed in the pharynx, tentacle bulb and the muscle that connects the two tentacle bulbs

We determined expression of *MlNanos1* and *2*, *MlVasa1* and *MlPiwi1* during the first 24–36 h of development to the cydippid stage (Fig. 2). We were not able to detect expression of the second nanos gene, *MlNanos2*, during any stage of development. *MlNanos1* was expressed maternally in a uniform pattern and uniformly through cleavage stages. Just prior to gastrulation (2–3 hpf), its expression was concentrated in cells around the blastopore and ectodermal cells at the aboral pole. These aboral ectodermal cells eventually form the polster cells of the comb rows. At 10–12 hpf, in addition to the comb row and pharynx staining, *MlNanos1* expression was also detected in the periphery of the tentacle bulbs. *MlVasa1* transcripts were not detected until after gastrulation, at about 7 hpf. Expression was detected around the blastopore, in the mesoderm, as well as an ectodermal domain in the sagittal plane. At 10–12 hpf, there was ectodermal expression adjacent to the comb rows (but not the polster cells themselves), as well as pharyngeal and tentacular expression. *MlPiwi1* was expressed uniformly in the egg through cleavage stages and gastrulation. After gastrulation, expression was in the ectoderm adjacent to the pharynx, mesodermal derivatives and four other unidentified groups of cells. At 10–12 hpf, expression is confined to the pharynx, tentacular muscle and the tentacle bulb apparatus, mostly the internal portion.

**Fig. 3:**
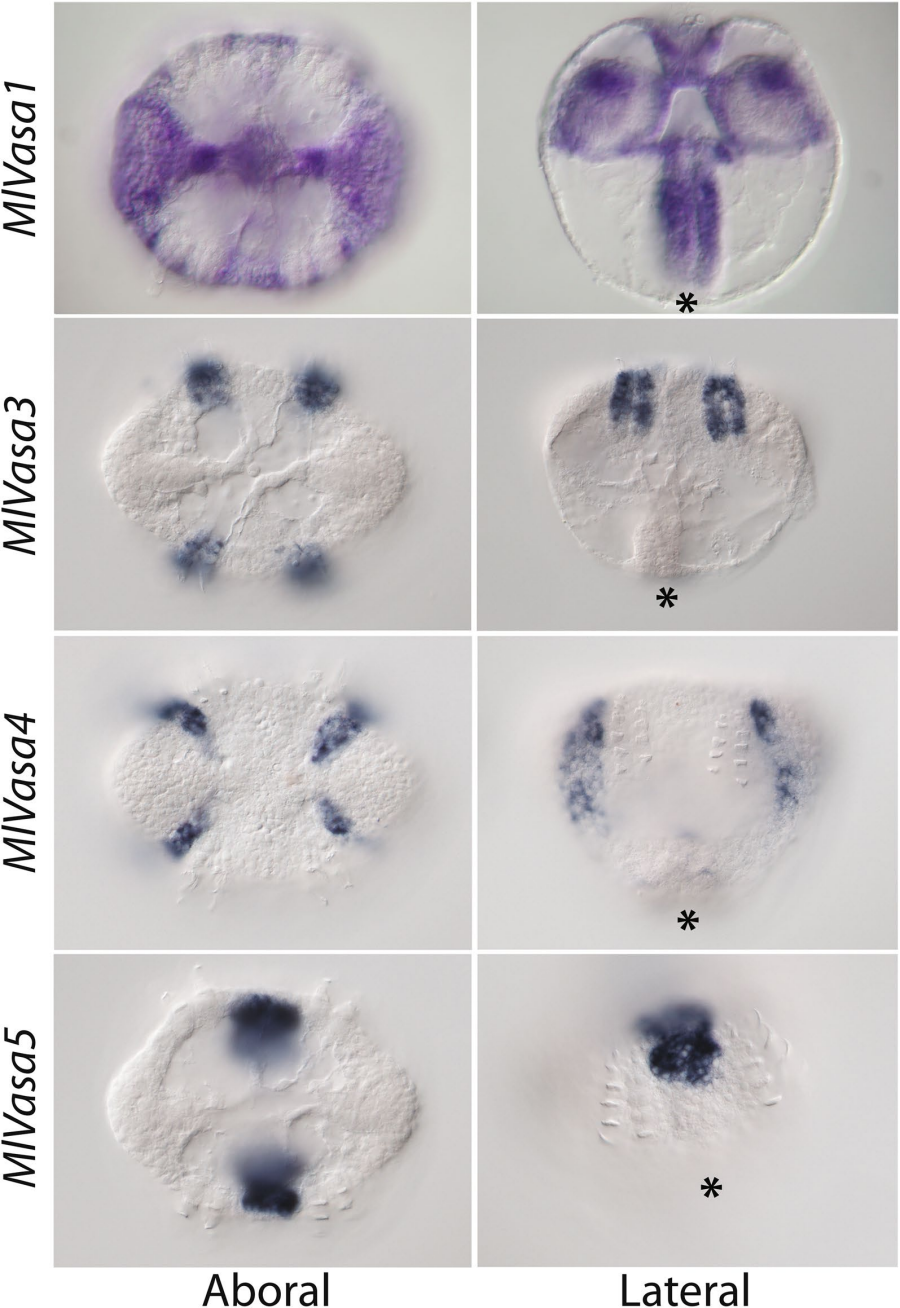
Expression of four vasa paralogs in *Mnemiopsis* embryos at 10–12 hpf. Views for each gene are shown in aboral (*left column*) and lateral (*right column*) orientation. *MlVasa1* was expressed in the ectoderm between the comb plates and the sagittal axis as well as in the tentacle bubs and pharynx. *MlVasa3* was expressed in the developing comb plates. *MlVasa4* also showed expression in the ectoderm between the comb rows and the tentacle bulbs. *MlVasa5* expression was detected aboral ectoderm in a region corresponding to the apical organ floor and the polar fields. Expression for *MlVasa2* was not determined

Our phylogenetic analyses showed that vasa underwent a lineage-specific expansion in ctenophores, with five copies present in *Mnemiopsis*. To determine whether these paralogs have unique expression domains during development, we compared their expression in embryos 10–12 hpf (Fig. 3). We detected expression of four of these (*MlVasa1* and *3*–*5*) that each had a unique expression domain, three of which were largely associated with different locations relative to the developing comb rows. Whereas *MlVasa1* was expressed between the comb plates and the sagittal axis, *MlVasa3* was expressed in the developing comb plates and *MlVasa4* also showed expression in the ectoderm between the comb rows and the tentacle bulbs. *MlVasa5* expression was only observed in aboral region corresponding to the apical organ floor and the polar fields.

**Fig. 4:**
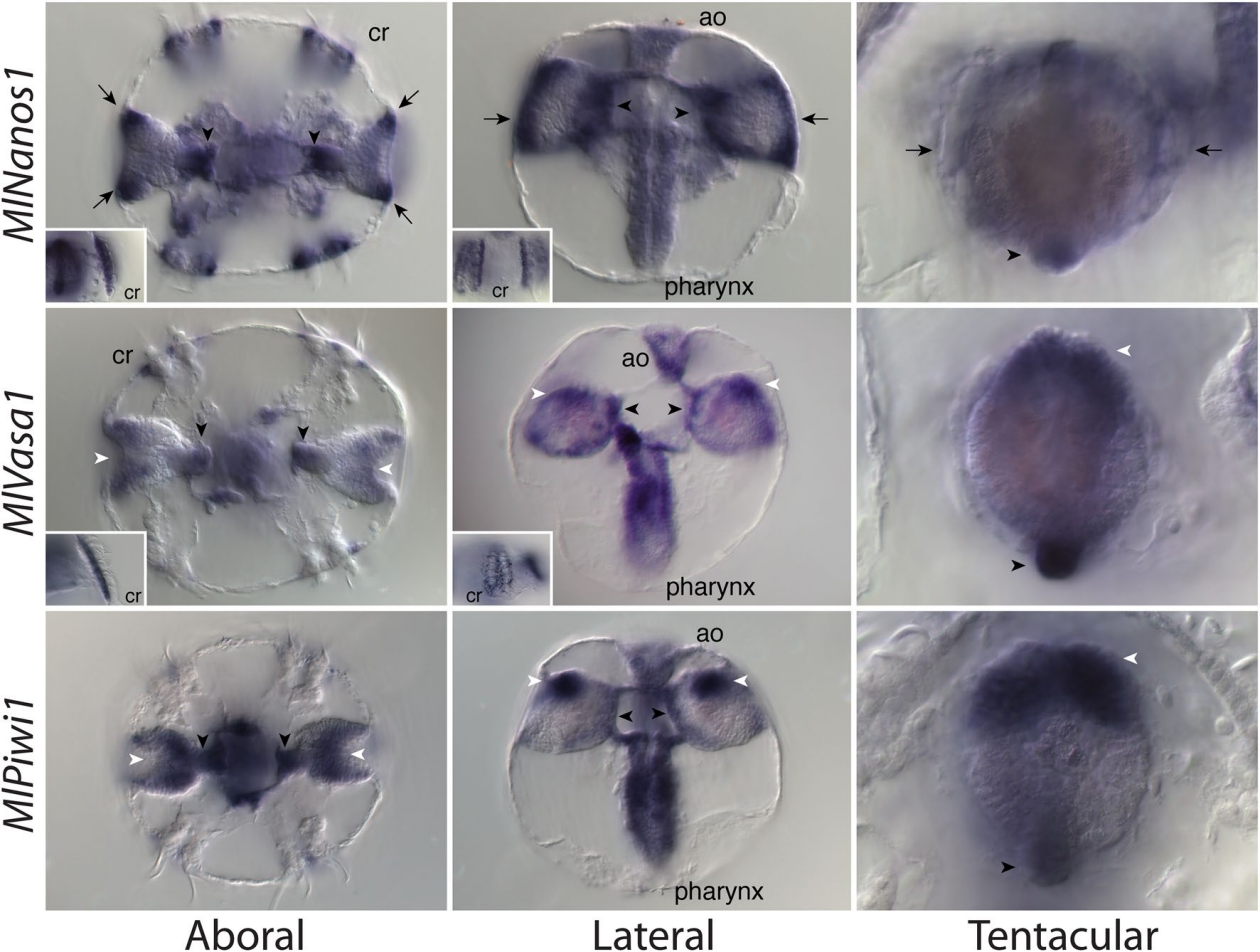
Expression of nanos, vasa and piwi genes in cydippid stages, approximately 18–24 hpf. *(Top row)* *MlNanos1* remained expressed in the comb rows (cr), as well as in two parts of the tentacle bulb, an interior portion (*black arrowheads*), as well as a region surrounding the exterior (*black arrows*). (*Middle row*) *MlVasa1* was expressed in cells adjacent to the comb rows (cr), different from that of *MlNanos1*. There was also expression detected in the interior part of the tentacle bulb (*black arrowhead*) similar to that of *MlNanos1* and an additional domain in the aboral part of the tentacle bulb (*white arrowhead*). (*Bottom row*) *MlPiwi1* was expressed in similar tentacular domains as *MlVasa1* (*white arrowhead*), as well as in the pharynx, and muscle connecting the tentacle bulbs (*black arrowhead*)

During cydippid stages (18–24 hpf), there was complementary expression of *MlNanos1* and *MlVasa1* associated with the comb rows (Fig. 4). Similar to the developmental expression, *MlVasa1* was expressed in ectodermal cells adjacent to and in between the comb rows, whereas *MlNanos1* was expressed in the polster cells themselves. *MlPiwi1* was expressed in the tentacular domains as well as the pharynx and muscle connecting the tentacular bulbs. *MlNanos1*, *MlVasa1* and *MlPiwi1* had two areas of overlapping gene expression along the entire length of the pharynx and the apical organ. In the tentacle apparati, there was also overlapping expression of all three genes. All three genes are expressed in an internal domain, the endodermal portion of the tentacle bulb (black arrowheads). Along the external side of the tentacle apparatus, *MlVasa1* and *MlPiwi1* are expressed primarily in a domain at the aboral extremity (white arrowheads), while *MlNanos1* was expressed more laterally (black arrows). We did not detect significant levels of expression of any of these genes in regions of the ctenophore where future gonadal regions are positioned.

**Fig. 5:**
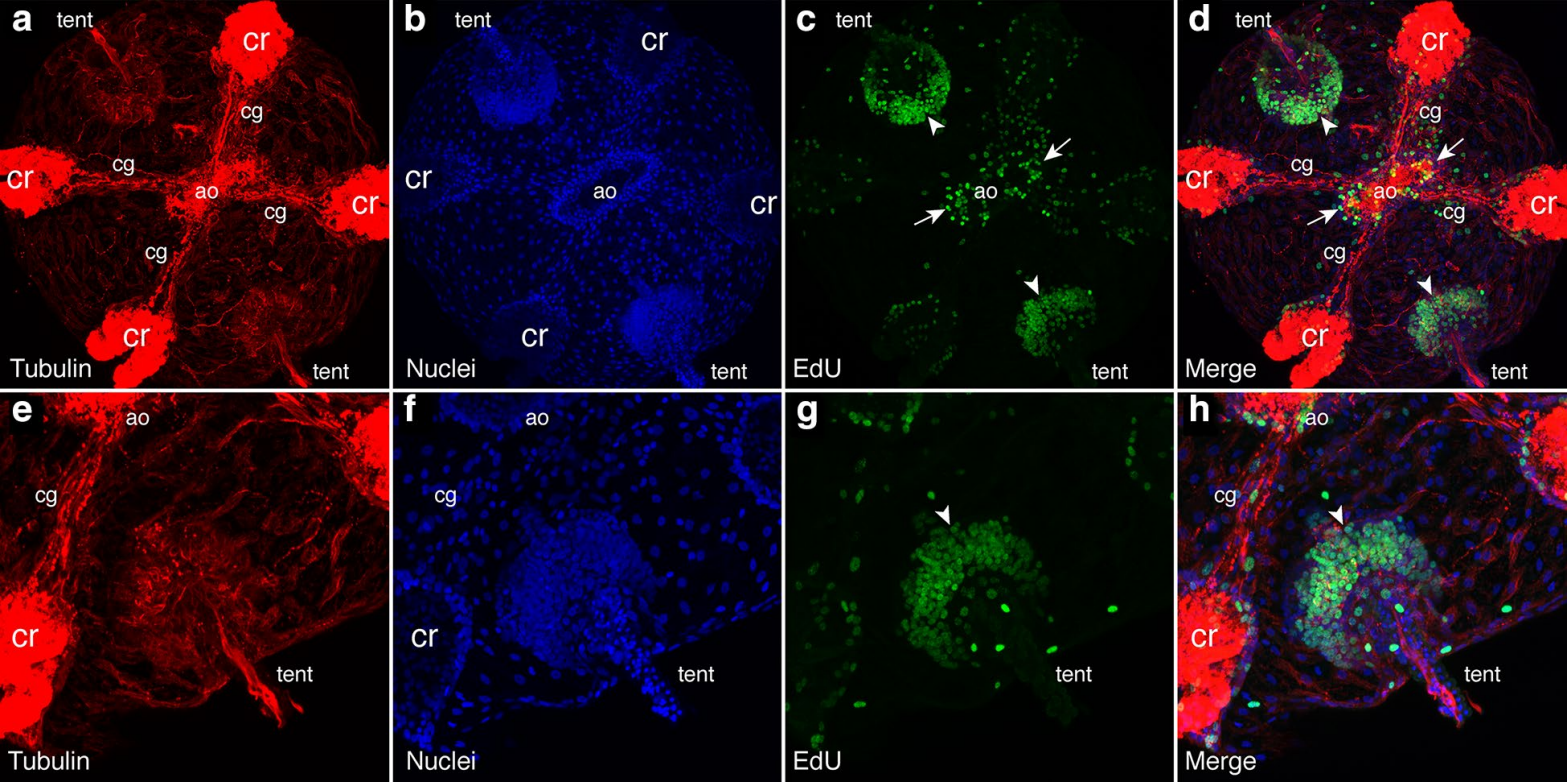
Confocal projections of EdU labeling of *Mnemiopsis* cydippids (18–24 hpf) to assay for cell proliferation. **a**–**d** Confocal projection of aboral portion of cydippid stage labeled with anti-tyrosine tubulin in *red* (**a**), which stains the nervous system as well as the cilia of the ciliated groove (cg) and comb rows (cr), Hoechst-stained nuclei in *blue* (**b**), EdU-labeled nuclei in *green* (**c**) and the merged (**d**). The apical organ (ao) was at the center, along with the two tentacle bulbs (tent) adjacent. *White arrows* show EdU labeling in the apical organ floor, and *white arrowheads* show labeling in the tentacle bulb apparati. **e**–**h** Same embryo as in **a**–**d** zoomed in close to the tentacle bulb

A previous study with *Pleurobrachia* had shown that vasa and piwi genes were expressed in regions of high mitotic activity [62], suggesting that these genes are expressed in “stem cells” or cells that are undergoing rapid proliferation. To determine areas of cell proliferation in cydippid stages, we utilized the Click-iT EdU labeling and detection kit. We observed two areas that had high incorporation of EdU, the apical organ and the tentacle bulbs (Fig. 5). In the apical organ, there was extensive proliferation in the apical organ floor (white arrows), similar to the expression patterns of *MlNanos1*, *MlVasa1* and *MlPiwi1*. Additionally, there are high levels of proliferation in the tentacle bulb apparati (white arrowheads), in similar locations as putative stem cell gene expression. We observed minimal levels of labeling in the epidermis and in the pharynx (data not shown).

**Fig. 6:**
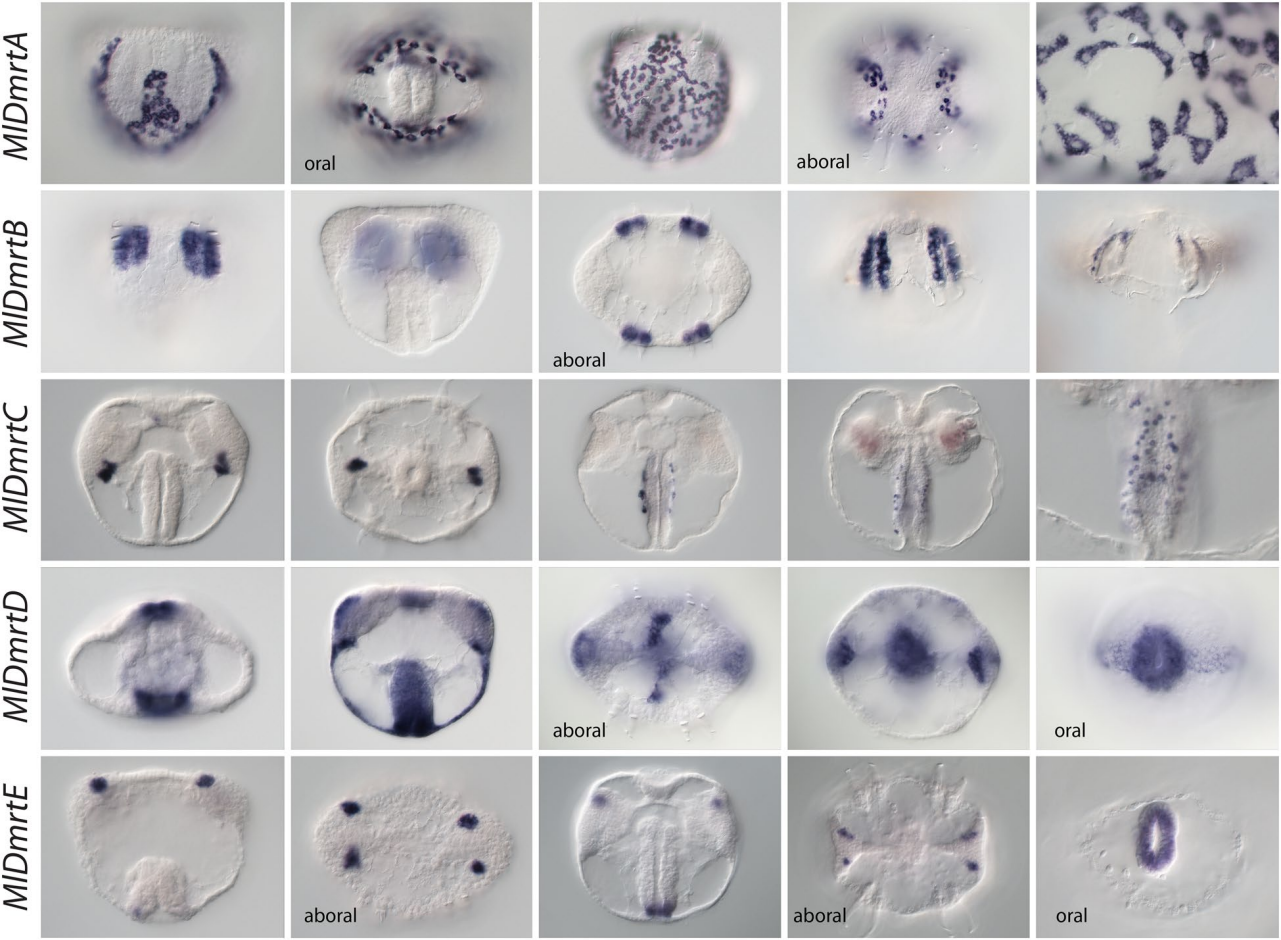
Expression of all 5 Dmrt genes during development of *Mnemiopsis*. (*1st row*) *MlDmrtA* was broadly expressed in the ectoderm outside of the developing comb rows, mouth and aboral organ. (*2nd row*) *MlDmrtB* was specifically expressed in the four developing comb rows. (*3rd row*) MlDmrtC was initially detected at the base of the tentacle bulbs with later expression in diffuse cells lining the pharynx in the cydippid larvae. (*4th row*) *MlDmrtD* was expressed in the oral and aboral regions of the gastrulating embryo, where expression remained in the cydippid stage. Expression was also observed in the tentacle buds, apical organ and an ectodermal band extending from the mouth. (*5th row*) *MlDmrtE* expression was initially observed in four regions of the gastrulating embryo, which continued into the cydippid stage, and later in the mouth

**Fig. 7:**
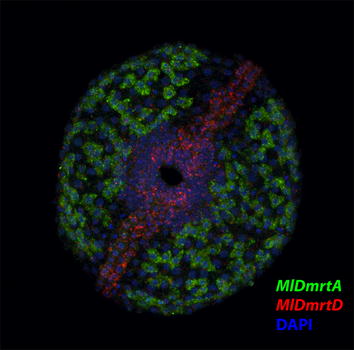
Double in situ of *MlDmrtA* and *MlDmrtD* in embryos 10–12 hpf showing adjacent expression in the oral ectoderm for these two Dmrt genes

Expression of the five Dmrt genes for *Mnemiopsis* (Fig. 6) was not detected until at least the gastrulation stage (7–9 hpf). *MlDmrtA* was broadly expressed in the ectoderm outside of regions of the tentacle bulbs and the comb rows, potentially corresponding to gland cells due to the number of positive cells, although the morphology also resembled neurons. Transcripts were diffuse and broadly distributed throughout the cytoplasm in a subset of these ectodermal cells. We did not observe any *MlDmrtA* expression in apical organ, which contains numerous neurons, suggesting these cells were not neurons. *MlDmrtB* showed expression specifically in the four pairs of developing comb rows. Efforts to differentiate expression of the two isoforms with separate riboprobes synthesized from the splice variants failed to result in discernable expression between these isoforms (data not shown). Expression of *MlDmrtC* was initially observed in two groups of cells at the base of the tentacle bulb apparatus. These cells then appeared to migrate to the inner lining of the pharynx later in development and distribute throughout the pharynx. During early gastrulation, *MlDmrtD* was expressed in endomesoderm near the blastopore and aboral pole, with later expression throughout the pharynx and portions of the tentacle buds. Expression remained concentrated around the mouth and aboral pole up until the cydippid stage. There was also an additional expression domain in the ectoderm, around the mouth and along the tentacular plane. Comparisons of expression of *MlDmrtD* with *MlDmrtA* suggested non-overlapping expression domains in the ectoderm. A double in situ hybridization with each gene further supported the single gene results where these two Dmrt genes have exclusionary domains (Fig. 7). Lastly, *MlDmrtE* was initially detected in the early gastrula stage as four distinct patches of cells in the tentacle bulb, potentially sensory cells. Expression in these four groups was maintained into the cydippid stage. *MlDmrtE* was also detected surrounding the mouth, similar to *MlDmrtD* at this developmental stage.

## Discussion

The genes nanos, vasa, piwi and Dmrt appear to be restricted to metazoans and thus animal novelties, whereas other genes such as *Magonashi*, *Tudor* and *Pumilio* have members in other eukaryotes [63, 70]. While vasa is only found in metazoans, there are numerous other eukaryotic DEAD box RNA helicases, including PL-10, that are very closely related to vasa and found more broadly in eukaryotes [69]. Among the non-bilaterians, the sponge *Amphimedon* has orthologs of all these genes (except for Dmrt), but potential Dmrt genes have been identified in the sponges *Sycon ciliatum* and *Oscarella carmela* [53]. Ctenophores [62] and cnidarians [e.g., 6, 19, 38, 71] also have orthologs of all of these genes. The only inferred loss from *Mnemiopsis* we have detected in our analysis is the potential lineage-specific loss of piwi ortholog in the piwiA group. However, the absence of a ctenophore gene in this group may reflect extensive sequence divergence in the currently annotated group containing only ctenophore genes (*MlPiwi1*, *3*, *4*; *PpiPiwi1*) or, if ctenophores represent the first branch of the animal kingdom, piwiA-type genes may have evolved after ancestor of ctenophores and the rest of animals. Of animals currently surveyed, only *Trichoplax* lacks vasa and piwi orthologs, which is interesting because placozoans have only been observed to reproduce asexually. Sexual reproduction has not been directly observed in placozoans; however, putative embryos have been observed and molecular signatures suggest that sexual reproduction does occur [72]. Whether these genes were lost in this lineage or never present in the lineage depends on the phylogenetic placement of *Trichoplax*. Considering that nearly all molecular phylogenies have them branching after sponges and ctenophores, this would suggest that these genes were lost in *Trichoplax*.

In this study, the expression of a suite of “germline” genes in the development of *Mnemiopsis* suggests that most of these markers are expressed in somatic cells or proliferative cells through development to the cydippid stage. While many of these genes are expressed early, none of them appear to be segregated to the endomesodermal lineage, from which the germ cells are thought to be descended from, or the future location of the male or female gonads along the meridional canals, beneath the comb rows. The latest development stage we have assayed is the cydippid larval stage, which is typically immature. However, for *Mnemiopsis*, previous research has shown individuals will undergo a pedomorphic transition termed dissogeny, where the cydippid stage will produce mature gametes, suggesting that the germline is likely to be specified quite early in development [58]. The only gene we studied that may correspond to this gametogenic location is *MlVasa3*; however, the expression was predominately ectodermal. Most of these genes are expressed in proliferative cells, which suggest that rather than being “germ cell” markers, perhaps these genes are more indicative of stem cells during development. It should be noted, as discussed in the Introduction, that in most of the other animals studied, these genes are expressed in cell types other than germ cells, which supports hypothesis and abundance of data that these genes play other multiple roles during development, particularly in stem cells [e.g., 62, 63, 73]. Indeed, in the leech *Helobdella*, vasa, piwi and nanos showed broad expression in somatic cell populations throughout embryogenesis and later have a second phase of expression in the male or female germline [74]. Like *Mnemiopsis*, *Helobdella* is also a hermaphrodite, which may explain the differential and potential delayed expression of these germline markers, or that PGCs are specified later and migrate to the future gonadal region. What is interesting here though is that in *Mnemiopsis*, they also have many non-overlapping expression patterns during development, which suggests that there may be several different kinds of “stem cells.” Currently, we do not know what genes are expressed or when in the germ cell progenitors for *Mnemiopsis*. Previous results from *Pleurobrachia* showed that Sox genes are also expressed in gonads. Sox2/12, like vasa and piwi, is expressed in the *Pleurobrachia* gametogenic regions and gametes; however, early developmental expression is not known [62, 75]. *Mnemiopsis* Sox2 is not expressed in a gametogenic region during early development [76], further suggesting that either *Mnemiopsis* germ cells are differentiated later than the cydippid stage, germ cells migrate to the future gonadal region, or differences in expression of germline-associated genes between these two ctenophore species. Comparisons of expression for these genes in reproductively mature *Mnemiopsis* would clearly be of interest to determine whether they are expressed in reproductive adults.

Little is known regarding ctenophore “stem cells.” Previous research has shown the basal part of the tentacle bulb is where the cells of the tentacle originate [77]. Because the tentacle constantly grows throughout the life of the animal, a continuous supply of cells is necessary. It is also known that different regions of the tentacle bulb give rise to different cell types, with the medial region giving rise to muscle and nerve cells, while the flanking regions give rise to the sticky colloblast cells [77]. The gene expression reported here shows that this area of the tentacle bulb expresses *MlNanos1*, *MlVasa1* and *MlPiwi1*, as well as displays high levels of cell proliferation, which does support the hypothesis that these are stem cells or progenitor cells. A previous study of the expression of Sox genes, a gene family also commonly expressed in stem cells, in *Mnemiopsis* showed all five of the measured Sox genes were expressed in tentacle bulbs and other regions with high cell proliferation [76]. Additionally, areas of high cell proliferation in *Pleurobrachia* were associated with the expression of piwi and vasa as well as Sox genes. Members of the vasa and piwi family are expressed in multiple regions of the adult stage of *Pleurobrachia* (early embryological stages were not studied), including comb rows, tentacle buds and gametogenic regions [62]. These genes may be maintaining these cells in an undifferentiated state and/or regulating cell proliferation and renewal. The somatic expression of these genes in the vasa, nanos, piwi and Sox families may also be a reason for the regenerative capacity of ctenophores. *Mnemiopsis* is known to be able to regenerate even when large portions of their body are removed [78, 79]. Perhaps these other populations of *MlNanos1*-, *MlVasa1*- and *MlPiwi1*-expressing cells are similar to neoblasts in planarians and function in regeneration of lost tissue.

Our analysis of transcript splicing and developmental expression of Dmrt genes in *Mnemiopsis* suggests a combination of shared (i.e., splicing) and diverged (i.e., spatial expression) features for this gene family in ctenophores when compared with bilaterians. Broadly, Dmrt genes are best known as contributors toward sex-specific characteristics and can promote the phenotypes of either males or females in diverse bilaterians [40]. However, Dmrt genes are increasingly known to have functions outside of sex determination and/or sex differentiation [51, 53]. One common feature of Dmrt genes is differential splicing, which in some species is a central mechanism for sex determination. For example, in *Drosophila* the Dmrt gene *doublesex* (dsx) is named because it plays a role in determining both males and females [43]. Male and female fruit flies express dsx transcripts, but produce different isoforms via alternate splicing (DSX^M^ and DSX^F^) [43]. Indeed, splicing of Dmrt transcripts appears to be common in many animals, but the function and commonality of splice variants outside of sex determination in insects and other animals remain little known [41]. Through sequencing we identified two *Mnemiopsis* Dmrt genes with differential splicing. A spliced form of *MlDmrtA* resulted in a protein with a partial DM domain that would not bind DNA. *MlDmrtB* also had two splice forms; however, both resulting proteins had complete, but different DM domains. Due to substantial differences in the resulting DM domains for each protein, the two isoforms of *MlDmrtB* would likely bind divergent DNA sequence motifs and thus regulate different downstream genes. Interestingly, *MlDmrtB* is the only Dmrt gene expressed near the gametogenic region. We were unable to differentiate expression domains with riboprobes generated from RACE products from which we identified these isoforms, likely due to large amounts of shared sequence. Future research using more specific probes or using exon-specific quantitative PCR probes may determine whether isoforms have different temporal or spatial expression.

*Mnemiopsis*, like most all ctenophores, is hermaphroditic. Thus, the only region of an individual which would be classified as sex specific would be the testes and ovaries that are paired along the meridional canals. The expression of the five *Mnemiopsis* Dmrt genes revealed different discrete expressions during development. With the possible exception of *MlDmrtB*, which was expressed in the comb rows, none of these had expression consistent with a role in differentiation or development of gametogenic regions. However, as discussed above for piwi, vasa and nanos expression, it is currently not certain when PGCs develop and the gametogenic regions differentiate. The Dmrt genes were expressed in non-overlapping regions of the ectoderm (*MlDmrtA* and *MlDmrtD*), pharynx (*MlDmrtC*), mouth (*MlDmrtD* and *MlDmrtE*) and potential sensory cells in the tentacle buds (*MlDmrtE*). The only other hermaphrodites where Dmrt genes have been studied are the reef building coral *Acropora millepora* [48] and the planarian *Schmidteamediterranea* [80]. *A. millepora* DM 1 was found to be highly expressed in the tips of adult corals during the reproductive season. Because this coral is a simultaneous hermaphrodite and samples were collected from whole fragments, increased expression of this single Dmrt gene could not be determined if specific to gametogenic tissue or, further, to either sperm or oocytes. Cnidarians, like ctenophores, have numerous Dmrt genes with diverse expression patterns [49], and thus, it remains unclear the role for Dmrt genes in early diverging phyla. *Smed*-*dmd*-*1* has a male-specific role in *S. mediterranea* for development and maintenance of germ cells as well as accessory structures and is also expressed in neurons. Additional future studies that determine the function, if any, of Dmrt genes in sex determination/differentiation of species in early diverging animal phyla are essential to understand the antiquity of this gene family’s role in animal sex determination and sex differentiation.

## Conclusion

In this study we show that the ctenophore *Mnemiopsisleidyi* has multiple genes in the vasa, nanos, piwi and Dmrt gene families, many of which appear to be lineage-specific expansions in the ctenophore lineage. Most of these genes are expressed during development in specific structures or regions of the embryo, but we observe little evidence that any are expressed in the future location for gametes. Our observations are consistent with a hypothesis that *Mnemiopsis* germ cells develop by epigenesis, not preformation. There is the lack of expression of vasa, nanos and piwi in these future gametogenic regions of this species, but abundant expression in zones of cell proliferation supports a hypothesis that these “germline” markers are expressed in potential somatic stem cell populations during early development. Finally, Dmrt genes in *Mnemiopsis* were expressed in discrete, largely non-overlapping locations of the embryo suggesting diverse roles in development with uncertain potential functions in differentiation of gametes.

## Additional files

10.1186/s13227-016-0051-9 *Mnemiopsis* genes identified and reported in this study. **Table S2.** Primer sequences used for gene amplification and RACE PCR in this study.
10.1186/s13227-016-0051-9 Bayesian analyses of vasa and other closely related DEAD box RNA helicases, with *Mnemiopsis* genes marked with asterisks. Displayed is a consensus tree from four independent runs of two million generations, with posterior probabilities at each node. **Figure S2.** Bayesian analysis of piwi, argonaute, and argonaute-like RNA binding proteins, with *Mnemiopsis* genes marked with asterisks. **Figure S3.** Bayesian analysis of metazoan nanos zinc fingers. *Mnemiopsis* genes (*nanos1* and *nanos2*) are marked with asterisks. Phylogenetic analyses using maximum likelihood yielded similar results (not shown).

## Abbreviations

BCIP: 5-bromo-4-chloro-3-indolyl phosphate
CCHC: Cys–Cys–His–Cys
Dmrt: doublesex/mab-3-related transcription factor
hpf: hours postfertilization
mRNA: messenger ribonucleic acid
NBT: nitro-blue tetrazolium
NOAA: National Oceanic and Atmospheric Administration
RACE: rapid amplification of cDNA ends
