# Supplementary material for: Developmental expression of “germline”- and “sex determination”-related genes in the ctenophore Mnemiopsisleidyi
Source: EvoDevo. 2016 Aug 2;7:17. doi: 10.1186/s13227-016-0051-9 (PMC4971632; doi:10.1186/s13227-016-0051-9)
Supplement: Supplementary file 1 — 10.1186/s13227-016-0051-9 Mnemiopsis genes identified and reported in this study. Table S2. Primer sequences used for gene amplification and RACE PCR in this study. [file 13227_2016_51_MOESM1_ESM.docx]

Table S1. *Mnemiopsis* genes identified and reported in this study.

| Gene Name | Genome Accession |
| --- | --- |
| MlNanos1 | ML130210a |
| MlNanos2 | ML22086a |
| MlVasa1 | ML04714a |
| MlVasa2 | ML46086a |
| MlVasa3 | ML375915a |
| MlVasa4 | ML06931a |
| MlVasa5 | ML068317a |
| MlPL10 | ML00995a |
| MlPiwi1 | ML074233a |
| MlPiwi2 | ML14972a |
| MlPiwi3 | ML6017 |
| MlPiwi4 | ML009119a |
| MlAgo-like | ML310319a |
| MlDmrtA | ML017911a* |
| MlDmrtB | ML027318a |
| MlDmrtC | ML008118a |
| MlDmrtD | ML087212a |
| MlDmrtE | ML116814a |

*This gene model lacks one exon (exon 4) identified in both RACE PCR amplicons and in assembled transcripts as part of the genome database. See Figure 1 and associated text.

Table S2. Primer sequences used for gene amplification and RACE PCR in this study.

| Gene Name | Primers: Forward (5’ – 3’) | Primers: Reverse (5’ – 3’) |
| --- | --- | --- |
| MlVasa1 | 1:GACCCCAGACGCTATGGTTATCACACCG  2:CCACAAGGAGACCAGCAAGTTCGCC | 1:GGCGAACTTGCTGGTCTCCTTGTGG  2:TCGGGTCGGTGTGATAACCATAGCGTC |
| MlNanos1 | 1:TCTACTCGACCCACGCCCTGAAGTC  2:AAGTCCCCCGACGGTAAAGTCACG | 1:TCGTAAGCGAAGAGGATGGGACACG  2:CGTGACTTTACCGTCGGGGGACTTC |
| MlNanos2 | 1:CGGAGAAGATTCTAGTGTGTACACAACTCACAC  2:GATGTCAACTGCAGGAAAAGTCATATGCCC | 1:GGCAAAAAGAATTGGGCATATGACTTTTCCTGC  2:CCTGCAGTTGACATCAAAGTGTGAGTTGTG |
| MlPiwi1 | 1:TGGCAGTCAAGGAGCAGGAGTTGAGAGAC  2:AACCGCTCTTTCAAGACTCCCCACATCGC | 1:TGGATTCTCTTTGTCACGACCACGAACACC  2:TGGGGAGTCTTGAAAGAGCGGTTTTGATGTC |
| MlPiwi2 | TTCAGCGAGGGCGAAGGAAACAGAAG | ACACAACACAAGCAGCCTCAAGCCAGAC |
| MlDmrtA | AAGCTTGTGCCAGATGTCG | GAGATGATGGGGCTGTTCTG |
| MlDmrtB | ACCAAAGACGCTCAGAAAGC | GTCGATGTCATTCGTGTTGG |
| MlDmrtC | ACGGGCTCATGGTCCTAAG | TGGGTTACTGCTCCAAAACC |
| MlDmrtE | TTTAGCTCCGAGGTGGAGAG | TTGTCTCCTGGTTGGAGGAC |
