# Supplementary figures and images for: Developmental expression of “germline”- and “sex determination”-related genes in the ctenophore Mnemiopsisleidyi
Source: EvoDevo. 2016 Aug 2;7:17. doi: 10.1186/s13227-016-0051-9 (PMC4971632; doi:10.1186/s13227-016-0051-9)

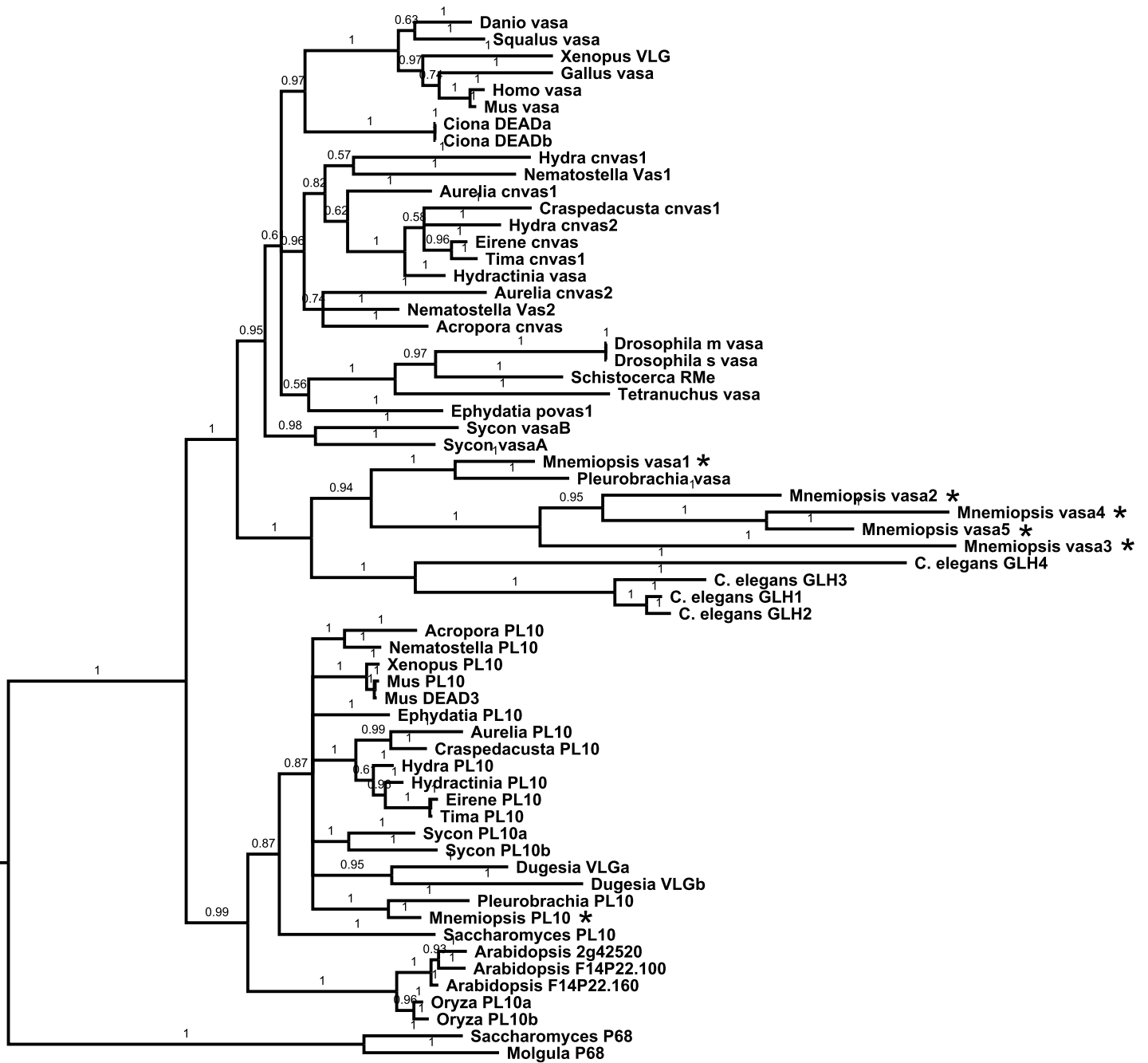

0.2

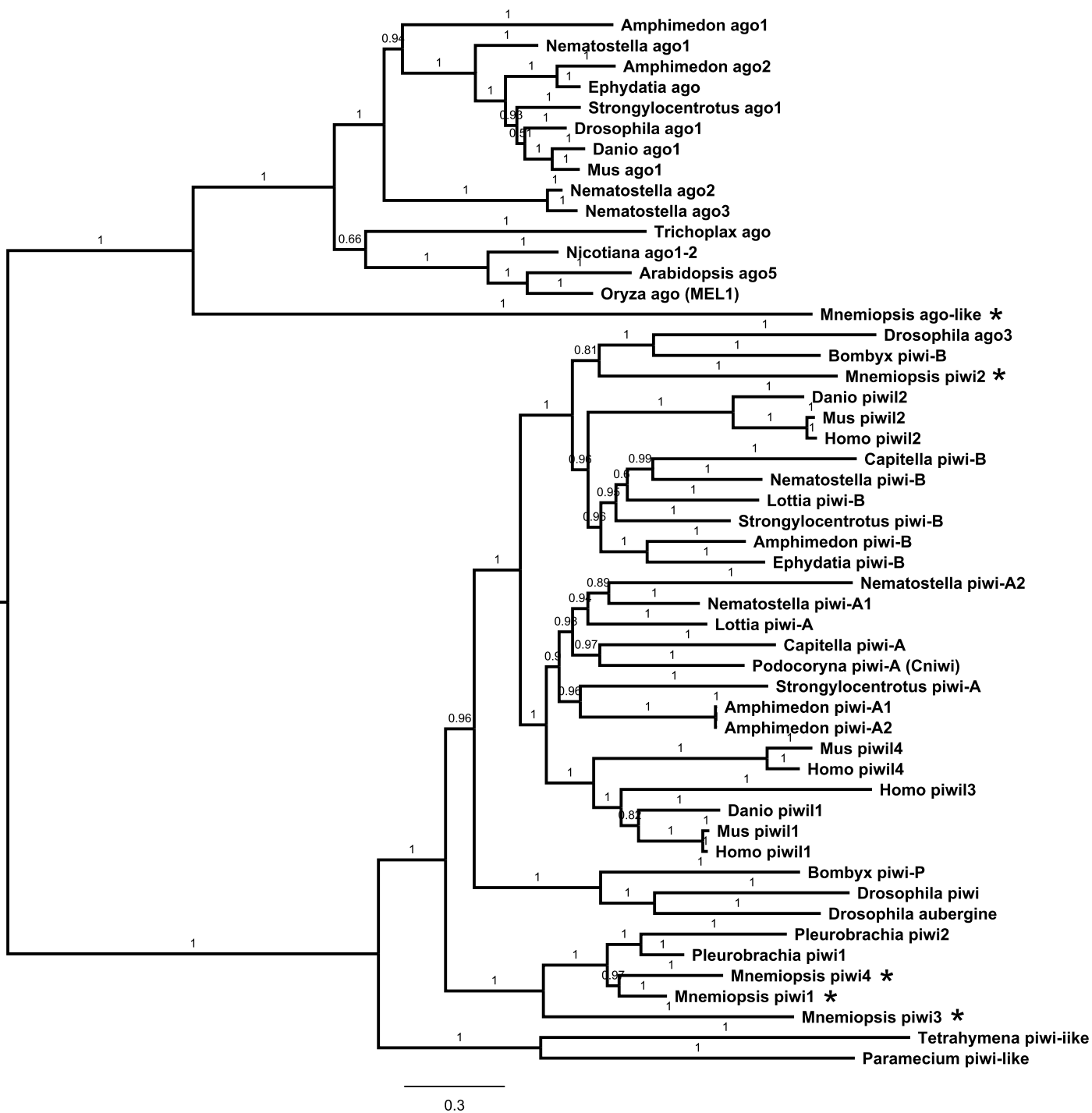

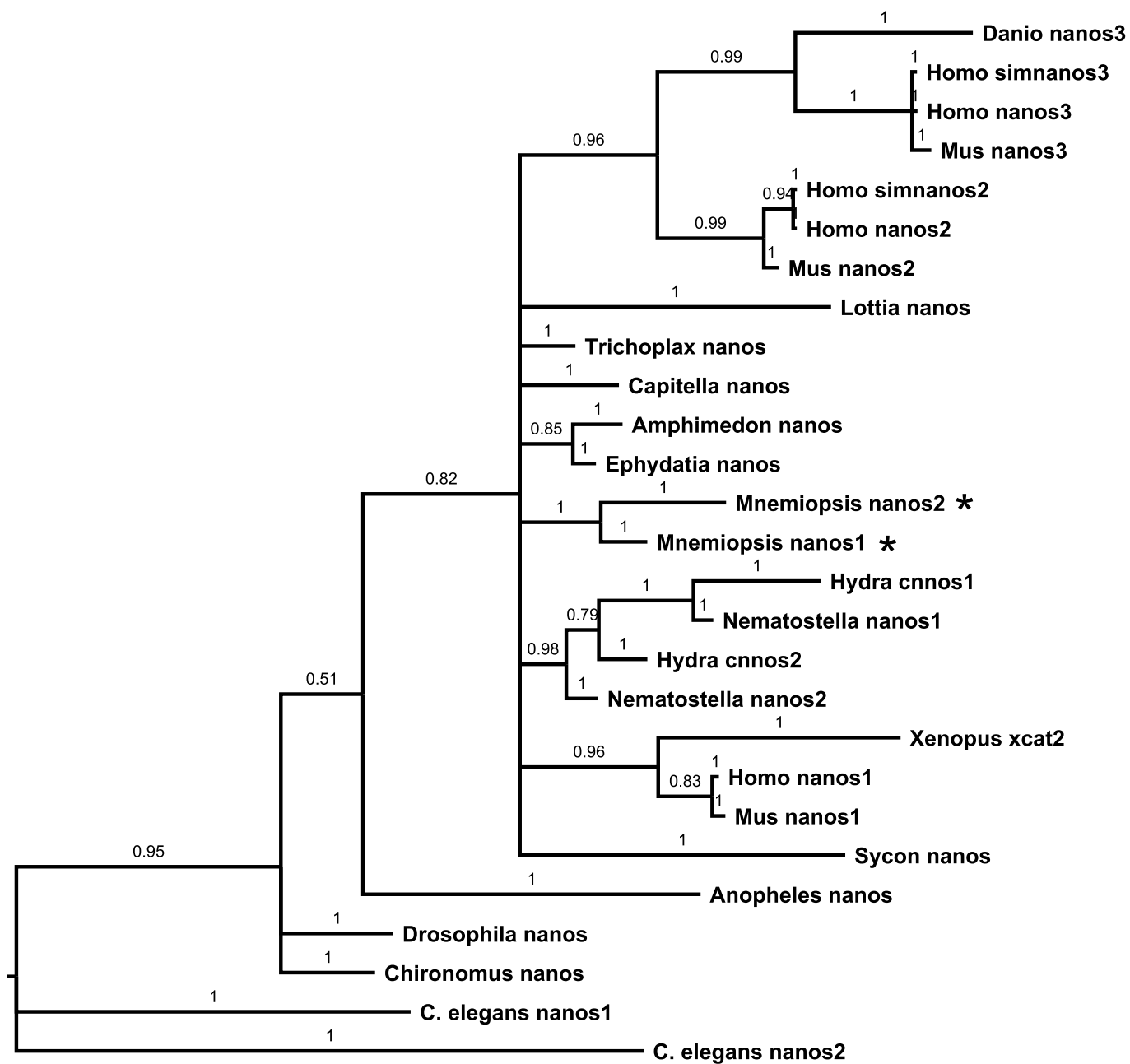

0.3

Supplement: Supplementary file 2 — 10.1186/s13227-016-0051-9 Bayesian analyses of vasa and other closely related DEAD box RNA helicases, with Mnemiopsis genes marked with asterisks. Displayed is a consensus tree from four independent runs of two million generations, with posterior probabilities at each node. Figure S2. Bayesian analysis of piwi, argonaute, and argonaute-like RNA binding proteins, with Mnemiopsis genes marked with asterisks. Figure S3. Bayesian analysis of metazoan nanos zinc fingers. Mnemiopsis genes (nanos1 and nanos2) are marked with asterisks. Phylogenetic analyses using maximum likelihood yielded similar results (not shown). [file 13227_2016_51_MOESM2_ESM.pdf]
